# Comparative glycomic analysis of *Mimiviridae* and *Marseilleviridae* uncovers host-related and lineage-specific glycosylation

**DOI:** 10.1093/jb/mvaf072

**Published:** 2025-12-09

**Authors:** Jinbo Shim, Chikako Hozumi, Masaki Kurogochi, Maho Yagi-Utsumi, Jun-ichi Furukawa, Masaharu Takemura, Hirokazu Yagi, Koichi Kato

**Affiliations:** Graduate School of Pharmaceutical Sciences, Nagoya City University, 3-1 Tanabe-dori, Mizuho-ku, Nagoya 467-8603, Japan; Exploratory Research Center on Life and Living Systems (ExCELLS), National Institutes of Natural Sciences, 5-1 Higashiyama, Myodaiji-cho, Okazaki 444-8787, Japan; Institute for Glyco-Core Research (iGCORE), Nagoya University, Furo-cho, Chikusa-ku, Nagoya 464-8601, Japan; Graduate School of Pharmaceutical Sciences, Nagoya City University, 3-1 Tanabe-dori, Mizuho-ku, Nagoya 467-8603, Japan; Exploratory Research Center on Life and Living Systems (ExCELLS), National Institutes of Natural Sciences, 5-1 Higashiyama, Myodaiji-cho, Okazaki 444-8787, Japan; Institute for Glyco-Core Research (iGCORE), Nagoya University, Furo-cho, Chikusa-ku, Nagoya 464-8601, Japan; Graduate School of Science, Tokyo University of Science, 1-3 Kagurazaka, Shinjuku-ku, Tokyo 162-8601, Japan; Graduate School of Pharmaceutical Sciences, Nagoya City University, 3-1 Tanabe-dori, Mizuho-ku, Nagoya 467-8603, Japan; Exploratory Research Center on Life and Living Systems (ExCELLS), National Institutes of Natural Sciences, 5-1 Higashiyama, Myodaiji-cho, Okazaki 444-8787, Japan; Graduate School of Pharmaceutical Sciences, Nagoya City University, 3-1 Tanabe-dori, Mizuho-ku, Nagoya 467-8603, Japan; Exploratory Research Center on Life and Living Systems (ExCELLS), National Institutes of Natural Sciences, 5-1 Higashiyama, Myodaiji-cho, Okazaki 444-8787, Japan

**Keywords:** giant virus, glycosylation*; Marseilleviridae*, mass spectrometry*; Mimiviridae*

## Abstract

Giant viruses encode unusual glycosylation machinery distinct from their amoebal hosts, raising fundamental questions about how their glycans are synthesized and diversified. Here, we present a comparative glycomic analysis of mimivirus, tokyovirus and hokutovirus, together with their common host *Acanthamoeba castellanii*. The main objective of this study was to determine whether giant viruses rely on host-derived *N*-glycosylation, or alternatively employ virus-encoded pathways to generate lineage-specific *O*-glycans, and to assess how these processes differ across virus families. *N*-glycan profiling revealed that all three viruses lack canonical eukaryotic core structures, in contrast to amoebal high-mannose *N*-glycans carrying pentose and phosphate residues. This finding demonstrates that giant viruses do not exploit the host secretory pathway for *N*-glycosylation, but instead depend on alternative mechanisms. *O*-glycan analyses showed lineage-specific patterns: family *Marseilleviridae* members tokyovirus and hokutovirus, displayed highly similar profiles, with minor virus-specific differences, whereas mimivirus exhibited structurally distinct glycans. Genomic inspection revealed that tokyovirus encodes only five glycosyltransferase-like genes, while *A. castellanii* harbours candidate enzymes for unusual monosaccharides. These findings clarify the distinct contributions of host and viral pathways and highlight evolutionary diversification of glycosylation among giant viruses.

## Graphical Abstract

**Graphical Abstract:**
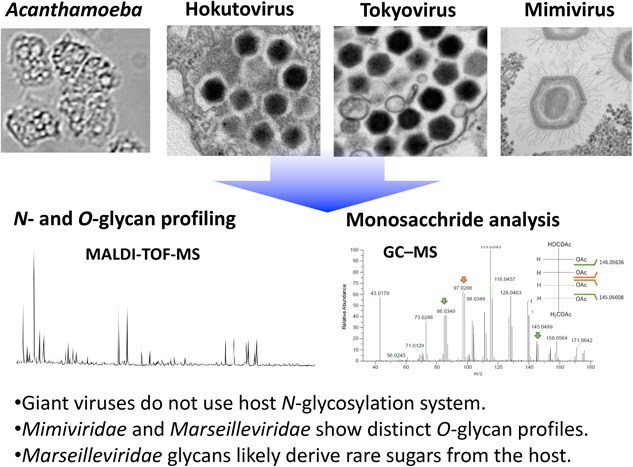

Giant viruses, including members of the families *Mimiviridae* and *Marseilleviridae*, are remarkable for their large particle sizes and complex genomes, in some cases exceeding those of many bacteria (1–5). These viruses infect amoebae and encode an unusually broad set of genes, including components typically associated with cellular life, such as DNA repair enzymes, translation factors, and, notably, glycosylation-related enzymes. The discovery of these metabolic genes has blurred the traditional boundaries between viruses and cellular organisms, suggesting that giant viruses may carry out biosynthetic functions rarely observed in other viral lineages.

Protein glycosylation is a fundamental post-translational modification that contributes to protein folding, stability, immune recognition and host–pathogen interactions (6–8). In eukaryotes, protein glycosylation occurs mainly as *N*-glycosylation, in which glycans with a conserved GlcNAc₂Man₃ core are attached to asparagine residues, and as *O*-glycosylation, in which diverse glycans are linked to serine or threonine residues (7). These two types exhibit conserved structural and physiological differences across eukaryotic species, reflecting their distinct roles in protein quality control, cell–cell communication and tissue protection (7). Whereas most viruses rely on host glycosylation pathways (9, 10), recent studies have revealed that certain giant viruses encode their own glycosyltransferases and other glycan biosynthetic enzymes, raising the possibility that they perform autonomous glycosylation (11–14). Nevertheless, the structural features, biosynthetic pathways and evolutionary origins of giant virus glycans remain poorly characterized.

Mimivirus has served as a model for viral glycosylation. It has reported that its major fibril protein is extensively glycosylated with long, complex *O*-linked glycans containing rare monosaccharides such as rhamnose, viosamine and GlcNAc (11, 15). More recently, virus-specific glycosyltransferases have been identified in both families *Mimiviridae* and *Marseilleviridae* genomes (Supplementary Table S1), suggesting that these enzymes synthesize lineage-specific glycans absent from the host (15, 16). Importantly, cryo-EM analyses of melbournevirus and tokyovirus revealed a glycosylated cap protein associated with the major capsid protein, demonstrating that *O*-linked glycosylation is also a conserved structural feature in the family *Marseilleviridae* (13, 14). Taken together, these findings highlight both the chemical diversity of mimivirus glycans and the presence of simpler, but evolutionarily conserved, *O*-glycans in *Marseilleviridae*.

Despite these advances comparative structural analyses of *N*- and *O*-linked glycans across multiple giant viruses and their amoebal hosts remain scarce. Moreover, the extent to which glycosylation patterns are conserved or diversified among phylogenetically related giant viruses has not been systematically examined.

Here, we report a comparative glycomic analysis of mimivirus, tokyovirus and hokutovirus—representing two major lineages of giant viruses—and their common amoebal host *Acanthamoeba castellanii*. Using MALDI-TOF-MS/MS for structural profiling and GC–MS for monosaccharide analysis, we characterized both *N*- and *O*-linked glycans, evaluated lineage-specific glycan signatures and assessed the degree of glycome conservation and diversification. Our findings provide new insights into the structural diversity, evolutionary patterns and potential functional roles of glycosylation in giant viruses.

## Materials and Methods

### Virus propagation and purification

 Mimivirus (*Mimivirus shirakomae*, isolated from Japanese water environments) was propagated in *A. castellanii* strain Neff, recently proposed the redescription as *A. terricola* (17), under standard conditions as described in the previous study (18, 19). Amoebae were cultured in PYG medium at 30°C and infected at a multiplicity of infection (MOI) of approximately 1. Infected cultures were harvested ~ 48 h post-infection. Cell debris was removed by low-speed centrifugation at 600 × *g* for 10 min at 4°C, and the resulting supernatant was collected. Viral particles were pelleted by centrifugation at 6,000 × *g* for 30 min at 4°C.

For lipid removal, the viral pellet was disrupted by repeated shearing through a 27-gauge syringe needle and subsequently incubated with 70% (v/v) acetonitrile at 4°C for 2 h. The suspension was centrifuged at 10,000 × *g* for 15 min at 4°C, and the resulting pellet containing purified virus particles was recovered. An aliquot of the preparation was subjected to protein quantification using the Bradford method. The final virus preparation was stored at −80°C until use.

Tokyovirus and hokutovirus, both members of the family *Marseilleviridae* (20, 21), were propagated in *A. castellanii* using the same procedure as for *Mimivirus* up to the point of cell debris removal. Due to their slower replication kinetics, infected cultures were harvested after ~96 h (approximately twice the duration required for m*imivirus*). Viral particles were pelleted at 8,000 × *g* for 30 min at 4°C. All subsequent purification, lipid-removal and protein quantification steps were performed as described for *mimivirus*.

### MALDI-TOF-MS analysis for *N*- and *O*-glycomics

Glycoprotein samples were denatured at 95°C for 5 min in 1% (w/v) SDS. After cooling, SDS was neutralized by adding NP-40 to a final concentration of 1% (v/v). *N*-glycans were enzymatically released with PNGase F at 37°C overnight (12–16 h), following standard protocols described in glycomics studies (22). The released glycans were separated from peptides and proteins by C18 reversed-phase pipette tip cleanup (ZipTip), vacuum-dried and subjected to reductive labelling with aminooxy-functionalized tryptophanylarginine methyl ester (AOWR) to enhance MALDI-TOF-MS ionization efficiency and detection sensitivity. Labelled glycans were desalted again using ZipTips, dried and reconstituted in 50% (v/v) acetonitrile containing 0.1% (v/v) TFA. Each sample was then mixed 1:1 with 2,5-dihydroxybenzoic acid (DHB) matrix solution and spotted onto a MALDI target plate. Mass spectra were acquired in positive-ion mode using a Bruker MALDI-TOF-MS instrument (model specified elsewhere).

For *O*-glycan analysis, glycopeptide solutions were treated using the β-elimination in the presence of PMP (BEP) protocol (23, 24). Under mild alkaline conditions, *O*-linked glycans were simultaneously released and derivatized with PMP reagent, effectively suppressing peeling side reactions. Reactions were neutralized with acetic acid, desalted using C18 ZipTips and vacuum-dried. The derivatized glycans were reconstituted in 50% (v/v) acetonitrile containing 0.1% (v/v) TFA, mixed 1:1 with DHB matrix and analyzed by MALDI-TOF-MS in positive-ion mode (Bruker instrument, model to be specified). Glycan yields were quantified from MALDI-TOF-MS signal intensities using an internal standard for relative quantification.

### Glycopeptide enrichment for GC–MS

For monosaccharide composition analysis by GC–MS, glycopeptides were enriched according to previous study (22). Purified viral glycoproteins (approximately 500 μg) were solubilized in PTS buffer containing 100 mM Tris–HCl (pH 8.0), 12 mM sodium deoxycholate and 12 mM sodium lauroyl sarcosinate. Proteins were reduced with 10 mM dithiothreitol (DTT) at 56°C for 30 min and alkylated with 20 mM iodoacetamide at room temperature in the dark for 30 min. The alkylation reaction was quenched by adding excess DTT. Samples were then diluted with 50 mM ammonium bicarbonate and digested overnight at 37°C with a Trypsin/Lys-C mixture at an enzyme-to-substrate ratio of 1:100 (w/w). The digestion was stopped by acidification with 0.5% (v/v) TFA, followed by centrifugation to remove any precipitates. The supernatant containing glycopeptides was collected and glycopeptides were further enriched by hydrophilic interaction chromatography (HILIC-HPLC). The enriched fractions were dried under vacuum and stored at −20°C until analysis.

### Monosaccharide composition analysis by GC–MS

The monosaccharide composition of viral and amoebal glycopeptides was determined using gas chromatography–mass spectrometry (GC–MS) following the previously described method with minor modifications (25, 26). Enriched glycopeptides corresponding to approximately 500 μg of glycoprotein were hydrolyzed in 2 M trifluoroacetic acid (TFA) at 120°C for 2 h. The hydrolyzed samples were dried under a stream of N_2_ gas, reduced with sodium borodeuteride and acetylated with acetic anhydride to generate alditol acetates. The derivatized samples were dissolved in acetone and analyzed using an Orbitrap Exploris GC and Trace 1600 GC system (Thermo Fisher Scientific) equipped with a TG-5SILMS column (30 m × 0.25 mm × 0.25 μm, Thermo Scientific). The samples were automatically injected in split mode with a split ratio of 5. Helium was used as the carrier gas at a constant flow rate of 1 ml/min. The oven program was set as follows: 120°C for 3 min, increased to 280°C at 10°C/min, and then to 320°C at 15°C/min. The transfer line temperature was set at 280°C. Electron ionization (EI) was performed at 70 eV with an ion source temperature of 250°C, and mass spectra were recorded over an *m/z* range of 40–700. In addition, chemical ionization (CI) was carried out using methane as a reagent gas at a flow rate of 1.5 ml/min, with an ion source temperature of 150°C, and spectra were recorded over an *m/z* range of 100–700.

Allo-inositol (3 nmol) was included as an internal standard in each analysis. Biological samples analyzed included *A. castellanii* as the amoebal host control, mimivirus, tokyovirus and hokutovirus. Standard monosaccharide mixtures were prepared containing xylose, glucose, *N*-acetylquinovosamine (QuiNAc), fucose, galactose, *N*-acetylglucosamine (GlcNAc), 3-deoxy-D-manno-oct-2-ulosonic acid (Kdo), rhamnose, mannose, *N*-acetylviosamine (VioNAc), arabinose, *N*-acetylfucosamine (FucNAc) and *N*-acetylgalactosamine (GalNAc), each at 3 nmol per injection. Each monosaccharide standard was also run individually to confirm retention times and mass spectral patterns. QuiNAc (Biosynth MA64786), Kdo (Biosynth MD04654), FucNAc (Biosynth MA15410), UDP-VioNAc were purchased from commercial suppliers. UDP-VioNAc was also obtained from a commercial source and hydrolyzed in 2 M trifluoroacetic acid (TFA) at 120°C for 2 h to generate VioNAc.

### Phylogenetic tree

Sequence alignments were generated using the ClustalW program in MEGA 12 (27). From this alignment, a maximum likelihood phylogenetic tree was constructed with 1,000 bootstrap replicates using substitution model of LG + G.

## Results

### Absence of canonical *N*-glycans in giant viruses

**Fig. 1:**
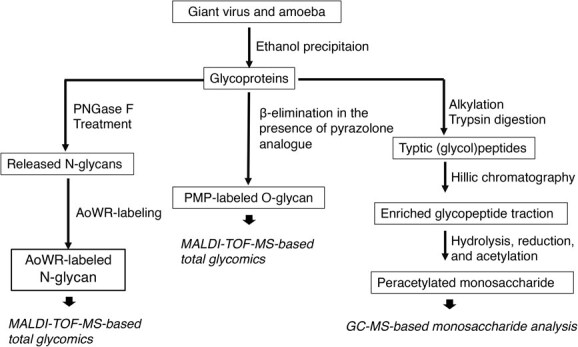
**Overview of analytical workflows for glycomics and glycoproteomics of host Acanthamoeba and giant viruses (tokyovirus, hokutovirus, and mimivirus)**. The left panel illustrates the *N*-glycomic workflow. Glycoproteins were digested with PNGase F to release *N*-glycans, which were subsequently labelled with 2-aminobenzoic acid derivative AoWR and analyzed by MALDI-TOF-MS. The middle panel depicts the *O*-glycomic workflow. *O*-glycans were released by β-elimination in the presence of a pyrazolone analogue (PMP), enabling simultaneous PMP labelling. PMP-labelled *O*-glycans were analyzed by MALDI-TOF-MS, and major peaks were further characterized by MS/MS to determine their monosaccharide composition and branching patterns. The right panel shows the monosaccharide analysis workflow. Glycoproteins were digested with trypsin, and the resulting glycopeptides were enriched using hydrophilic interaction chromatography (HILIC). Monosaccharide composition analysis was conducted using GC–MS.

**Fig. 2:**
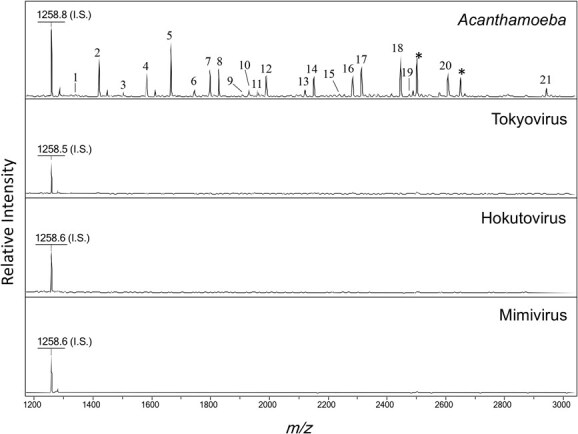
***N*-glycome profiling of glycoproteins derived from *Acanthamoeba* and giant viruses.** MALDI-TOF mass spectra of *N*-glycans were released by PNGase F digestion of glycoproteins from *Acanthamoeba* and giant viruses. The signal at 1,258 *m/z* corresponds to the internal standard (Hex_5_). When a composition could not be assigned based on Hex, HexNAc, dHex, Pen or + 80, it is indicated with an asterisk (*). Peak numbers correspond to those listed in Table I.

Protein fractions from giant virus and *A. castellanii* were treated with PNGase F to release *N*-glycans bearing canonical eukaryotic core structures, followed by AoWR labelling and MALDI-TOF-MS analysis (Fig. 1). By assigning compositions to the observed mass values, glycan structures were inferred (Fig. 2 and Table I). In *A. castellanii*, abundant high-mannose-type glycans decorated with pentose residues and phosphate group were detected, consistent with previously reported amoebal *N*-glycan profiles (28). In contrast, mimivirus, tokyovirus and hokutovirus showed no detectable *N*-glycans containing the canonical core structure.

**Table I:** Abundant *N*-glycans in *Acanthamoeba* strains

Peak	Observed mass (*m/z*)	Proposed sequence	Relative intensity
IS	1258.8	Hex_5_	100.0
1	1340.7	GN_2_M_3_	3.9
2	1420.8	GN_2_M_3_ + 80	56.1
3	1502.9	GN_2_M_3_ + Hex_1_	8.4
4	1582.9	GN_2_M_3_ + Hex_1_ + 80	38.7
5	1665.0	GN_2_M_3_ + Hex_2_	80.7
6	1745.0	GN_2_M_3_ + Hex_2_ + 80	11.0
7	1797.1	GN_2_M_3_ + Hex_2_ + Pen_1_	38.7
8	1827.1	GN_2_M_3_ + Hex_3_	39.4
9	1908.0	GN_2_M_3_ + Hex_3_ + 80	5.8
10	1929.1	GN_2_M_3_ + Hex_2_+ Pen_2_	11.0
11	1959.1	GN_2_M_3_ + Hex_3_ + Pen_1_	10.3
12	1989.1	GN_2_M_3_ + Hex_4_	32.3
13	2121.2	GN_2_M_3_ + Hex_3_ + Pen_1_	12.9
14	2151.2	GN_2_M_3_ + Hex_5_	32.3
15	2237.4	GN_2_M_3_ + Hex_2_ + Pen_2_ + dHex_1_ + Hex_1_	5.2
16	2283.2	GN_2_M_3_ + Hex_5_ + Pen_1_	32.3
17	2313.2	GN_2_M_3_ + Hex_6_	45.2
18	2445.3	GN_2_M_3_ + Hex_6_ + Pen_1_	58.1
19	2476.4	GN_2_M_3_ + Hex_7_	6.5
20	2607.4	GN_2_M_3_ + Hex_7_ + Pen_1_	38.7
21	2941.9	GN_2_M_3_ + Hex_7_ + Pen_2_ + HexNAc_1_	12.9

Peak numbers correspond to those shown in Fig. 2. The data from initial glycomic screening are summarized to show *m/z* values and the predicted compositions of the 21 major *N*-glycans derived from PNGase F digests of *Acanthamoeba*. Relative peak intensities of the major glycans were calculated, with the internal standard glycan normalized to 100. Glycan structures were predicted using GlycoMod, and only those with a calculated mass deviation of less than 0.5 Da from the observed *m/z* values were selected. GN_2_M_3_ denotes the canonical *N*-glycan core structure, consisting of two *N*-acetylglucosamine residues (GlcNAc; GN_2_) and three mannose residues (Man; M_3_). Suffixes indicate additional monosaccharide units or modifications: “Hex$\square$” represents *n* hexose residues (such as glucose, mannose or galactose), “HexNAc$\square$” represents *n N*-acetylhexosamine residues (such as GlcNAc or GalNAc), “Pen$\square$” represents *m* pentose residues (such as xylose, arabinose or ribose) and “dHex$\square$” represents *k* deoxyhexose residues (such as fucose or rhamnose). The notation “80” indicates an additional mass increment of 80 Da, typically corresponding to a phosphate group. In this table, the “+” symbol simply indicates compositional addition of the corresponding monosaccharide or modification to the core structure, GN_2_M_3_. Unless otherwise noted, all masses correspond to [M + H]^+^ adduct ions detected by MALDI-TOF-MS.

### *O*-glycan profiles reveal lineage-specific signatures

**Fig. 3:**
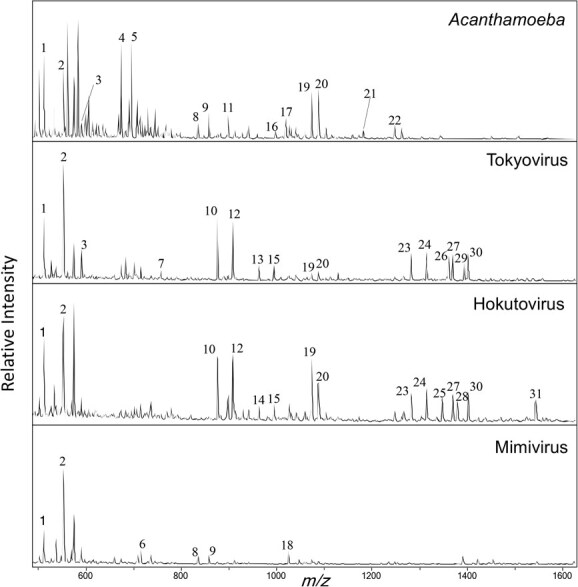
***O*-glycome profiling of glycoproteins derived from *Acanthamoeba* and giant viruses.** MALDI-TOF mass spectra of *O*-glycans were released by PMP-assisted β-elimination from Acanthamoeba and giant virus glycoproteins. Major peaks unique to each viral lineage are indicated, and their tentative compositions were deduced from MS/MS analysis. Peak numbers correspond to those listed in Table II.

**Fig. 4:**
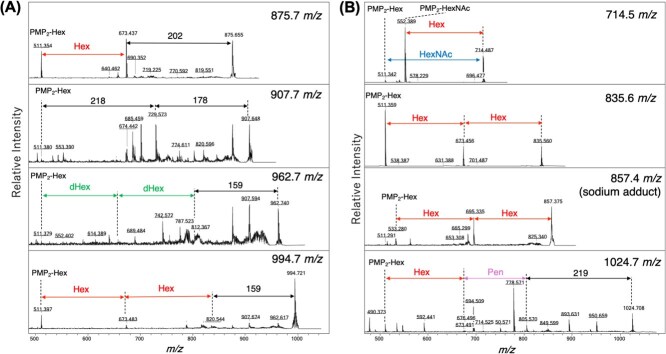
**Representative MS/MS spectra of PMP-labelled *O*-glycans from giant viruses.** Representative MS/MS spectra from (A) tokyovirus and (B) mimivirus show characteristic neutral-loss fragments. Precursor ions at *m/z* 875.5, 907.5, 962.5 and 994.5 yielded diagnostic product ions. Spectra were acquired in LIFT mode, and major fragment peaks are annotated with their *m/z* values.

*O*-glycans from giant viruses and *A. castellanii* were prepared using the PMP-assisted β-elimination method and analyzed by MALDI-TOF-MS/MS (Figs 3 and 4, and Supplementary Fig. S1). From 100 μg of starting protein material, the following total glycan yields were obtained:

*A. castellanii,* 107.3 pmol; tokyovirus, 56.4 pmol; hokutovirus, 50.7 pmol; and mimivirus, 86.4 pmol. Most detected glycans derived from giant viruses and *A. castellanii* consisted of HexNAc and Hex monosaccharides. Several peaks were specific to giant viruses and absent from the host. Within the family *Marseilleviridae*, tokyovirus and hokutovirus, showed highly similar *O*-glycan profiles, sharing peaks at *m/z* 876, 908, 963 and 995. MS/MS spectra indicated that these correspond to structures such as PMP₂-Hex-Hex-202, PMP₂-Hex-218-178, PMP₂-Hex-dHex-dHex-159 and PMP₂-Hex-Hex-Hex-159 (Fig. 4A). The diagnostic neutral losses of 159, 178, 202 and 218 Da suggest the incorporation of unusual monosaccharides, including deoxyamino sugars (*e.g.* perosamine), polyhydroxylated hexoses, amino-substituted HexNAc and Kdo-like residues, which are not typically found in canonical eukaryotic glycans.

These virus-specific peaks therefore provide direct evidence that *Marseilleviridae O*-glycans are decorated with chemically modified sugars absent from the host repertoire. In addition to the shared family *Marseilleviridae* profile, tokyovirus exhibited peaks unique to this virus at *m/z* 1316 and 1393, whereas hokutovirus displayed low-abundance but distinctive signals at *m/z* 1347 and 1543. Although the precise structural assignments of these peaks remain unresolved, their occurrence highlights potential lineage-specific differences in *O*-glycan composition. Moreover, MS/MS fragmentation patterns suggested that these glycans frequently contain pentose residues and may include a 187 Da monosaccharide consistent with a deoxyHexNAc unit.

Among the giant viruses analyzed, mimivirus exhibited a distinct glycomic profile compared with the family *Marseilleviridae* members. Mimivirus prominently expressed oligosaccharides composed of three or four consecutive hexose units (Fig. 4B), which were less evident in the other viruses. These findings are consistent with previous studies (29). Although similar glycans were also present in *A. castellanii*, our subsequent GC–MS analysis indicated that the hexose composition differs between host and virus, suggesting that the viral glycans are not identical to their host counterparts. Compositions predicted from MS and MS/MS spectra are summarized in Table II.

**Table II:** Comparative *O*-glycan of *A. castellanii*, tokyovirus, hokutovirus and mimivirus determined by MALDI-TOF-MS

	Observed mass (*m/z*)				Proposed sequence	Relative intensity			
Peak	Host	Tokyovirus	Hokutovirus	Mimivirus		Host	Tokyovirus	Hokutovirus	Mimivirus
1	511.2	511.3	511.3	511.3	PMP_2_ -Hex	120.0	55.2	78.6	36.8
2	552.3	552.3	552.3	552.4	PMP_2_ -HexNAc	100.0	100.0	100.0	100.0
3	591.2	591.3			PMP_2_ -Hex+80	25.0	29.9	-	-
4	673.4				PMP_2_ -Hex-Hex	138.3	-	-	-
5	695.3				PMP₂-Hex-Hex (sodium adduct)	140.0	-	-	-
6				714.5	PMP_2_ -HexNAc-Hex, PMP_2_ -Hex-HexNAc	-	-	-	15.8
7		756.5			PMP_2_ -HexNAc-HexNAc	-	11.5	-	-
8	835.5			835.6	PMP_2_ -Hex-Hex-Hex	23.3	-	-	9.5
9	857.5			857.5	PMP_2_ -Hex-Hex-Hex (sodium adduct)	40.0	-	-	11.6
10		875.7	875.7		PMP_2_ -Hex-Hex-202	-	54.0	61.9	-
11	898.5				PMP_2_ -Hex-Hex-HexNAc (sodium adduct)	36.7	-	-	-
12		907.6	907.7		PMP_2_ -Hex-218-178	-	51.7	64.3	-
13		962.7			PMP_2_ -Hex-dHex-dHex-159	-	13.8	-	-
14			962.7		PMP_2_ -Hex-dHex-159-dHex	-	-	15.0	-
15		994.7	994.7		PMP_2_ -Hex-Hex-Hex-159	-	14.9	17.9	-
16	997.6				PMP_2_ -Hex-Hex-Hex-Hex	13.3	-	-	-
17	1019.6				PMP_2_ -Hex-Hex-Hex-Hex (sodium adduct)	31.7	-	-	-
18				1024.7	PMP_2_ -Hex-Hex-Pen-219	-	-	-	13.7
19	1073.6	1073.7	1073.7		PMP_2_ -HexNAc-Hex-156-HexNAc	66.7	6.9	60.7	-
20	1087.6	1087.6	1087.8		PMP_2_ -methylHexNAc-Hex-156-HexNAc	73.3	9.2	38.1	-
21	1181.7				PMP_2_ -Hex-Hex-Hex-Hex-Hex (sodium adduct)	13.3	-	-	-
22	1247.7				PMP_2_ -Hex-HexNAc-HexNAc-Hex-dHex (sodium adduct)	20.0	-	-	-
23		1282.0	1282.0		PMP_2_ -Hex-HexNAc-HexNAc-218-dHex	-	25.3	28.6	-
24		1314.0	1314.0		PMP_2_ -Hex-HexNAc-218-HexNAc-178	-	26.4	32.1	-
25			1347.1		PMP_2_ -Hex-(dHex₂ + 178 + 187)-178	-	-	23.8	-
26		1361.1			PMP_2_ -Hex-(Pen₂ + HexNAc₂)-178	-	20.7	-	-
27		1369.1	1369.1		PMP_2_ -Hex-(Pen_2_ + 187 + 218)-187	-	23.0	28.6	-
28			1379.1		PMP_2_ -Hex-(Pen_3_ + dHex_2_)-178	-	-	19.0	-
29		1393.1			PMP_2_ -Hex-HexNAc-178-187-159-159	-	14.9	-	-
30		1401.0	1401.0		PMP_2_ -Hex-(Pen_3_ + dHex_2_)-178 (sodium adduct)	-	23.0	28.6	-
31			1543.3		PMP_2_ -Hex-(Pen_1_ + dHex_1_ + HexNAc_2_ + 187 + 187)-178	-	-	19.0	-

Peak numbers correspond to those shown in Fig. 3. Observed mass values correspond to PMP-derivatized glycans detected by MALDI-TOF-MS. Proposed sequences were inferred from MS/MS fragmentation patterns and neutral-loss analyses. Relative intensity represents the abundance of each glycan peak normalized to the most intense signal (PMP_2_-HexNAc) within each dataset. Assignments that could not be directly resolved from MS/MS spectra were made according to the following notation: glycans are abbreviated as (Hex)_a_(HexNAc)_b_(Pen)_c_(dHex)_d_(dHexNAc)_e_,(159)_f_,(178)_g_,(202)_h_,(218)_i_, where each unit is additively combined to represent the overall composition. Hex denotes a hexose (Δ*m/z*162), HexNAc denotes an *N*-acetylhexosamine (Δ*m/z* 203), Pen denotes a pentose (Δ*m/z* 132), dHex denotes a deoxyhexose (Δ*m/z* 146) and dHexNAc denotes a deoxyHexNAc (Δ*m/z* 187). Unless otherwise noted, all masses correspond to [M + H]^+^ adduct ions detected by MALDI-TOF-MS. In the MS/MS annotation, the “+” symbol indicates neutral losses detected in the fragmentation spectra, representing the losses of two or more sugar units when individual monosaccharide fragments were not observed.

### Monosaccharide composition by GC–MS

**Fig. 5:**
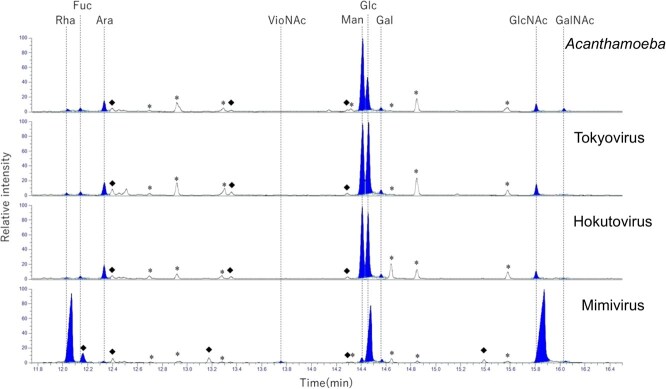
**Monosaccharide composition analysis of *Acanthamoeba* and giant virus peptides by GC–MS.** Monosaccharide compositions of glycoproteins from Acanthamoeba (host control), tokyovirus, hokutovirus and mimivirus were analyzed by GC–MS following acid hydrolysis, reduction and acetylation to generate alditol acetates. Chromatograms were acquired in CI mode, and peaks were identified by comparison with authentic monosaccharide standards. Detected monosaccharides included rhamnose (Rha), fucose (Fuc), arabinose (Ara), xylose (Xyl), mannose (Man), glucose (Glc), galactose (Gal), *N*-acetylviosamine (VioNAc), *N*-acetylglucosamine (GlcNAc) and *N*-acetylgalactosamine (GalNAc), assigned on the basis of retention times and mass spectra relative to authentic standards. The peaks corresponding to these monosaccharides were displayed as filled-in areas. Asterisks indicate byproducts of chemical reactions. The diamond denotes an unidentified monosaccharide-related compound, whose fragment pattern confirmed incorporation of deuterium derived from D₂O used in the reduction step.

To analyze monosaccharide composition, glycopeptides were reduced and subsequently acetylated to generate derivatized monosaccharides amenable to GC–MS analysis (Fig. 5). The resulting spectra revealed that tokyovirus, and hokutovirus are enriched in mannose and glucose as major hexoses. GlcNAc was predominantly detected among HexNAc species, along with a notable presence of arabinose. Minor components included rhamnose, fucose, arabinose and galactose, which are known constituents of amoebal host glycans. However, despite characteristic neutral losses such as 159, 178, 187, 202 and 218 Da observed in MALDI-TOF-MS/MS, the corresponding monosaccharides could not be definitively identified in the GC–MS spectra. Notably, mimivirus, in contrast to *Marseillevirus*, predominantly contained rhamnose, glucose, GlcNAc and VioNAc.

## Discussion

Our *N*-glycan analyses revealed that mimivirus, tokyovirus and hokutovirus completely lack glycans with the canonical eukaryotic core structure, in sharp contrast to the high-mannose *N*-glycans carrying pentose and phosphate residues observed in *A. castellanii* (28). Although many of their proteins contain putative *N*-glycosylation consensus motifs (Asn–X–Ser/Thr, where X ≠ Pro) (Supplementary Fig. S2), these motifs do not appear to be utilized for *N*-glycosylation in these giant viruses. These results provide direct evidence that these giant viruses do not exploit the host secretory *N*-glycosylation pathway for protein modification. Instead, their glycomes must be assembled through alternative routes, most likely driven by virus-encoded *O-*glycosylation machinery. Such a strategy distinguishes giant viruses from many enveloped viruses, such as influenza virus, which rely heavily on host-derived *N*-glycans for glycoprotein processing (9, 10).

It is considered that viral infection alters the physiological state of the host and thereby affects its endogenous glycan structures. Nevertheless, viral glycans are synthesized within cytoplasmic viral factories, spatially separated from the host Golgi apparatus where host *N*- and *O*-glycans are processed. Therefore, virus-specific glycans are unlikely to be incorporated into host glycoproteins.

For mimivirus, the glycan structures detected here are in good agreement with those previously report, where *O*-glycan analysis of the protein identified short oligosaccharides containing glucose (29). Our MALDI-TOF-MS/MS data reproduced these *O*-glycan signatures with high fidelity, raising the possibility that we are predominantly observing the basal oligosaccharide region attached directly to protein. By contrast, the long polysaccharide chains rich in rhamnose and GlcNAc described in earlier fibril studies were not evident in our MS/MS dataset, most likely because such extended chains fall outside the detection range of this method. Importantly, GC–MS profiling did reveal Rha- and GlcNAc-rich compositions, suggesting that the longer fibrillar polysaccharide chains may indeed be captured under conditions conductive to monosaccharide-level analysis (30).

In contrast, tokyovirus and hokutovirus showed no evidence of extended polysaccharide chains, a finding that is consistent with both electron microscopy observations and genomic analyses (20, 21). Comparative genomics has revealed clade-specific repertoires of glycosyltransferases in the families *Mimiviridae* and *Marseilleviridae*. Mimivirus encodes an expanded set of glycosylation enzymes, including putative rhamnosyltransferases and viosaminyltransferases, as well as other components of biosamine biosynthesis (Supplementary Table S1) (11, 15), whereas the family *Marseilleviridae* members such as tokyovirus harbor a reduced set (Supplementary Table S2). In tokyovirus, only five putative glycosyltransferase-like proteins have been annotated, three of which show highest homology to mannosyltransferases (Supplementary Table S2). Notably, subtle virus-specific differences between tokyovirus and hokutovirus were also detected, which may provide clues to lineage-specific diversification within the family *Marseilleviridae*.

MALDI-TOF-MS/MS analyses further revealed neutral-loss fragments at *m/z* 159, 178, 187, 202 and 218 Da, which cannot be explained by canonical Hex or HexNAc residues. Several plausible assignments can nevertheless be made. A 159 Da loss is consistent with the mass of perosamine (4-amino-4,6-dideoxyhexose), whose biosynthesis requires GDP-mannose 4,6-dehydratase (Gmd; KO K01711) and a PLP-dependent aminotransferase (PerA; KO K13010), with optional *N*-acetylation by PerB (KO K17939) (31, 32). A 178 Da loss may correspond to a polyhydroxylated hexose (C_6_H_10_O_6_), possibly generated by cytochrome P450 monooxygenases (CYP450 superfamily) or FAD-dependent oxidoreductases/hydroxylases (GMC oxidoreductase family) (33, 34). A 187 Da loss is compatible with deoxyHexNAc residues such as QuiNAc (2-acetamido-2,6-dideoxyglucose) or FucNAc (2-acetamido-2-deoxyfucose); however, GC–MS analyses using authentic standards did not yield a match, suggesting the presence of an alternative deoxyHexNAc isomer. Importantly, both the 159 Da (perosamine) and 187 Da (deoxyHexNAc) signals can be explained by the same Gmd–PerA–PerB enzymatic module acting on GDP-mannose intermediates. A 202 Da neutral loss neutral loss may correspond to an amino-substituted HexNAc, which could plausibly arise from the activity of PerA-like aminotransferase (KO K1310) (31). Finally, a 218 Da loss is consistent with the mass of Kdo (3-deoxy-D-manno-oct-2-ulosonic acid), whose biosynthesis in bacteria requires KdsA (KO K01627), KdsC (KO K07046) and KdsB (KO K00979) (35). Taken together, these observations strongly indicate that the unusual neutral losses detected in our MS/MS spectra originate from host-encoded pathways in *A. castellanii*, rather than from viral genes in tokyovirus. Specifically, tokyovirus does not encode Gmd, PerA/PerB, P450 monooxygenases, FAD-dependent oxidoreductases or KdsA/KdsC/KdsB, whereas homologs of these enzymes are present in the host genome of *A. castellanii* (Supplementary Table S3). In eukaryotic cells, cytochrome P450 monooxygenases are typically localized to the endoplasmic reticulum or mitochondria (36), whereas FAD-dependent oxidoreductases are mainly present in the cytosol, mitochondria or peroxisomes (37). During giant virus infection, host ER-derived membranes are reorganized into virus factories, which may bring these oxidative enzymes into close proximity with viral glycoprotein biosynthetic machinery. Such spatial reorganization could facilitate the selective hydroxylation of hexoses in viral glycans.

Taken together, the combined structural and genomic evidence supports a model in which tokyovirus and hokutovirus, as representatives of the family *Marseilleviridae*, produce mannose-rich *O*-glycans, reflecting a limited glycosylation system. By contrast, mimivirus of the family *Mimiviridae* encodes several glycosylation-related genes, yet the *O*-glycans detected in this study were comparatively simple. This suggests that our analyses primarily detected the basal glycan region directly attached to the protein, whereas more elaborate polysaccharide chains may lie outside the detection range of our methods. These findings highlight how divergent glycosylation strategies may have influenced the evolution and host interactions of giant viruses. Importantly, our study establishes a framework for comparative viral glycomics that can be extended to other clades, providing a foundation for future structural and evolutionary analyses. Whether the simpler glycomes of the family *Marseilleviridae* represent an ancestral state from which mimivirus expanded, or instead a secondary reduction from a more complex ancestral glycome, remains unresolved. Future studies integrating structural glycomics with comparative genomics across diverse giant virus clades will be required to clarify these evolutionary scenarios. The glycosyltransferase sequences of mimivirus and tokyovirus cluster separately from those of other giant viruses, reflecting lineage-specific evolution of glycosylation machinery (Supplementary Fig. S3). Our present work provides a foundational framework for comparative viral glycomics that can be extended to other clades, offering a basis for future structural and evolutionary analyses.

## Supplementary Material

Web_Material_mvaf072
