# Supplementary material for: Comparative glycomic analysis of Mimiviridae and Marseilleviridae uncovers host-related and lineage-specific glycosylation
Source: J Biochem. 2025 Dec 9;179(2):117–26. doi: 10.1093/jb/mvaf072 (PMC12866637; doi:10.1093/jb/mvaf072)
Supplement: Web_Material_mvaf072 [file web_material_mvaf072.zip › Supplementary data_shim et al.pdf]

Supplementary data

Supplementary Fig. 1:

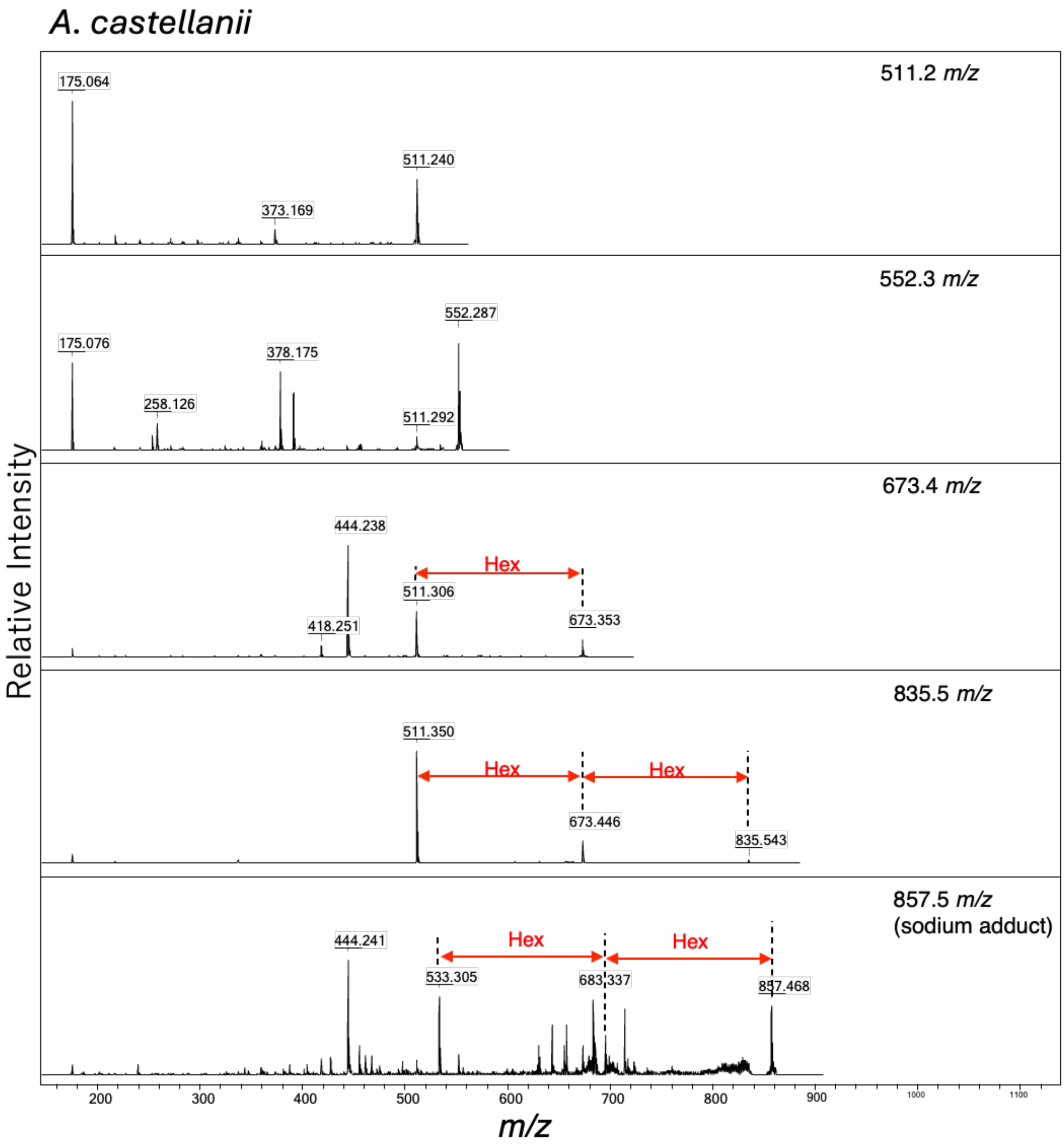

A. castellanii

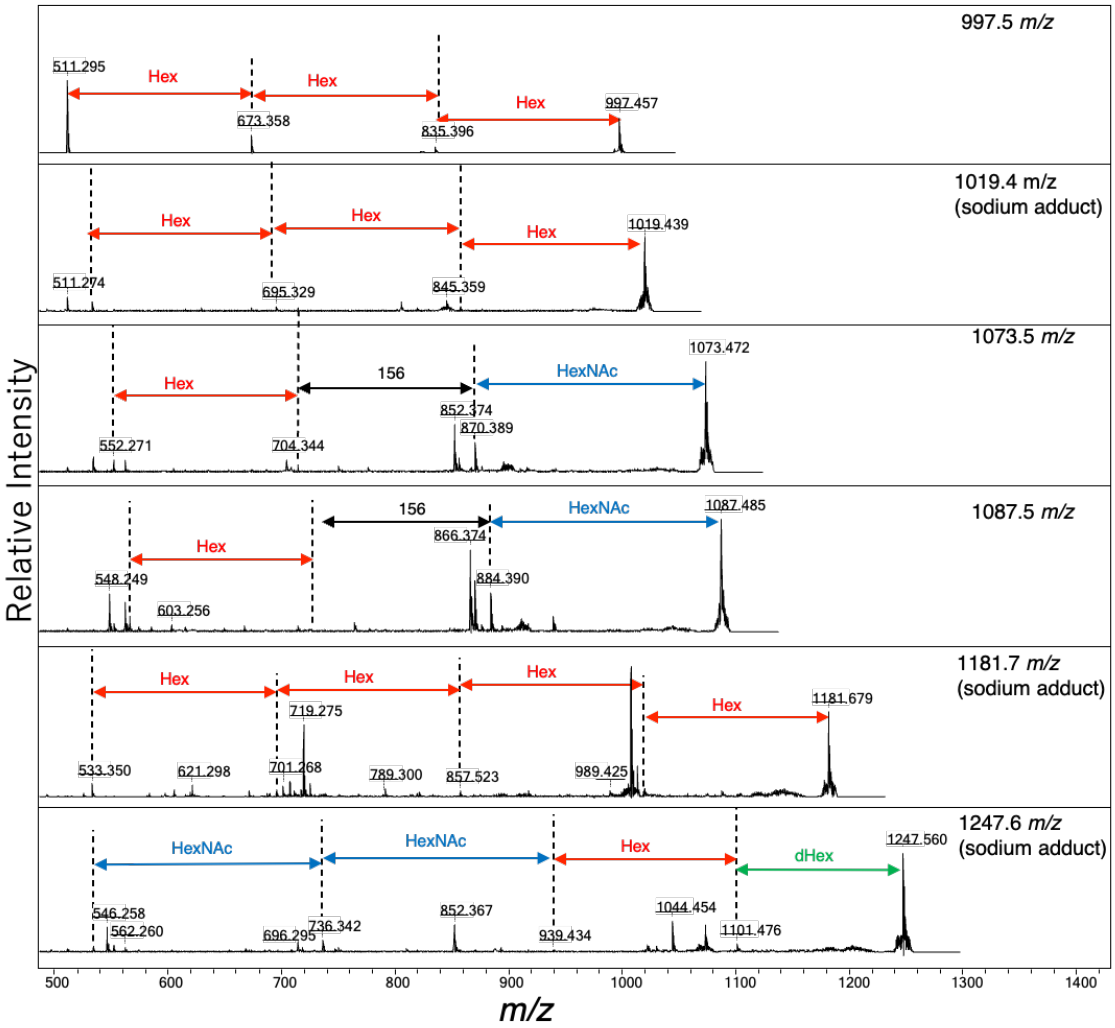

Tokyovirus

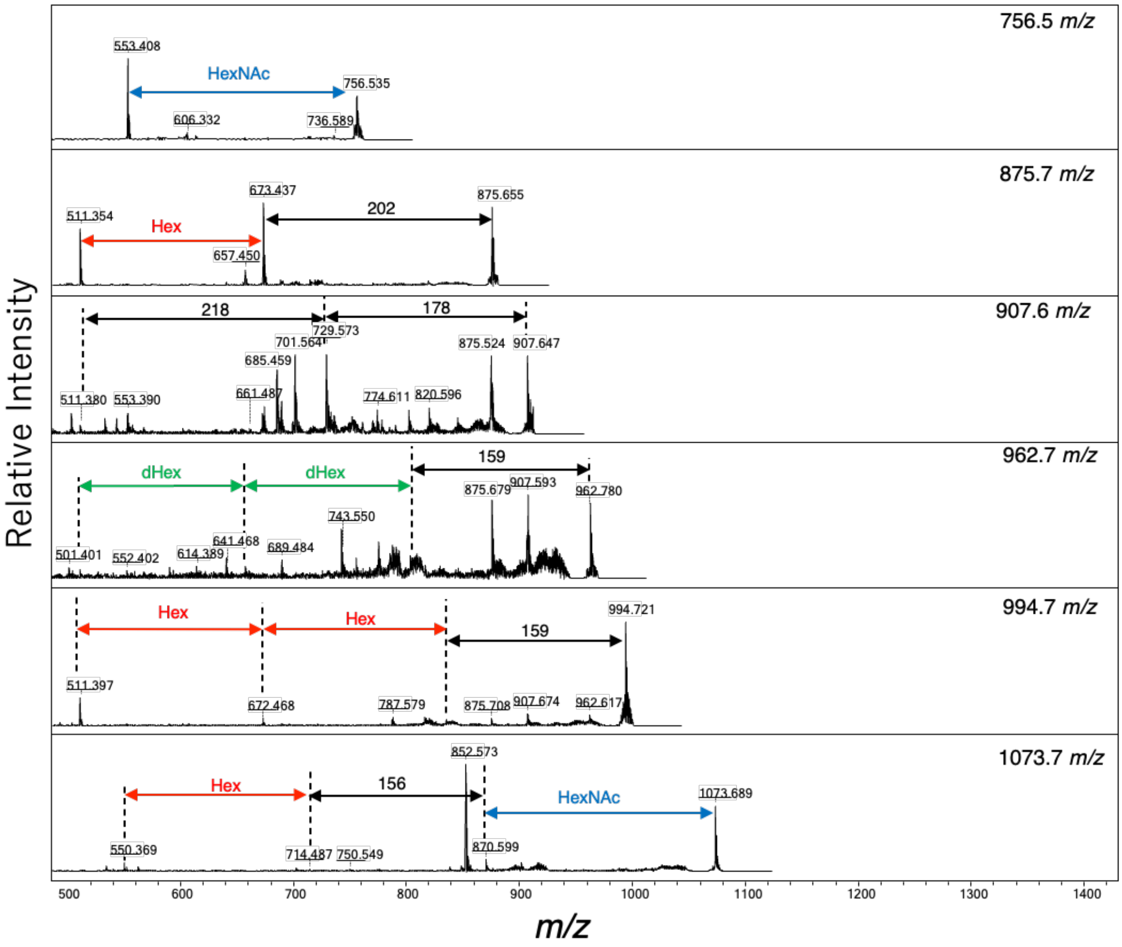

756.5 m/z
553.408
HexNAc
756.535
606.332
736.589
875.7 m/z
673.437
202
875.655
511.354
Hex
657.450
907.6 m/z
218
178
729.573
701.564
685.459
875.524
907.647
511.380
553.390
661.487
774.611
820.596
962.7 m/z
dHex
dHex
159
875.679
907.593
962.780
743.550
641.468
689.484
501.401
552.402
614.389
994.7 m/z
Hex
Hex
159
994.721
511.397
672.468
787.579
875.708
907.674
962.617
1073.7 m/z
852.573
Hex
156
HexNAc
1073.689
870.599
550.369
714.487
750.549
Relative Intensity
500
600
700
800
900
1000
1100
1200
1300
1400
m/z

Hokutovirus

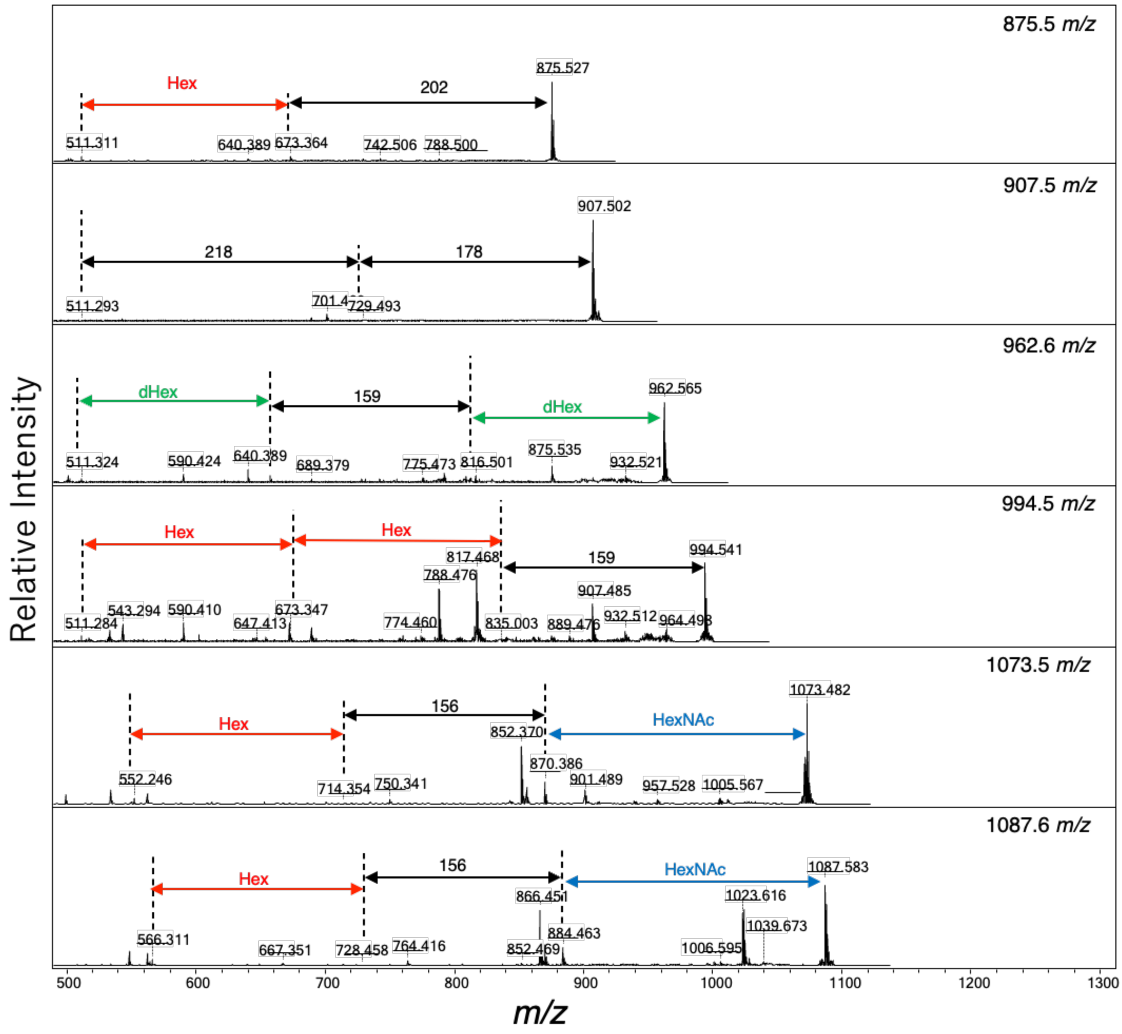

Hokutovirus

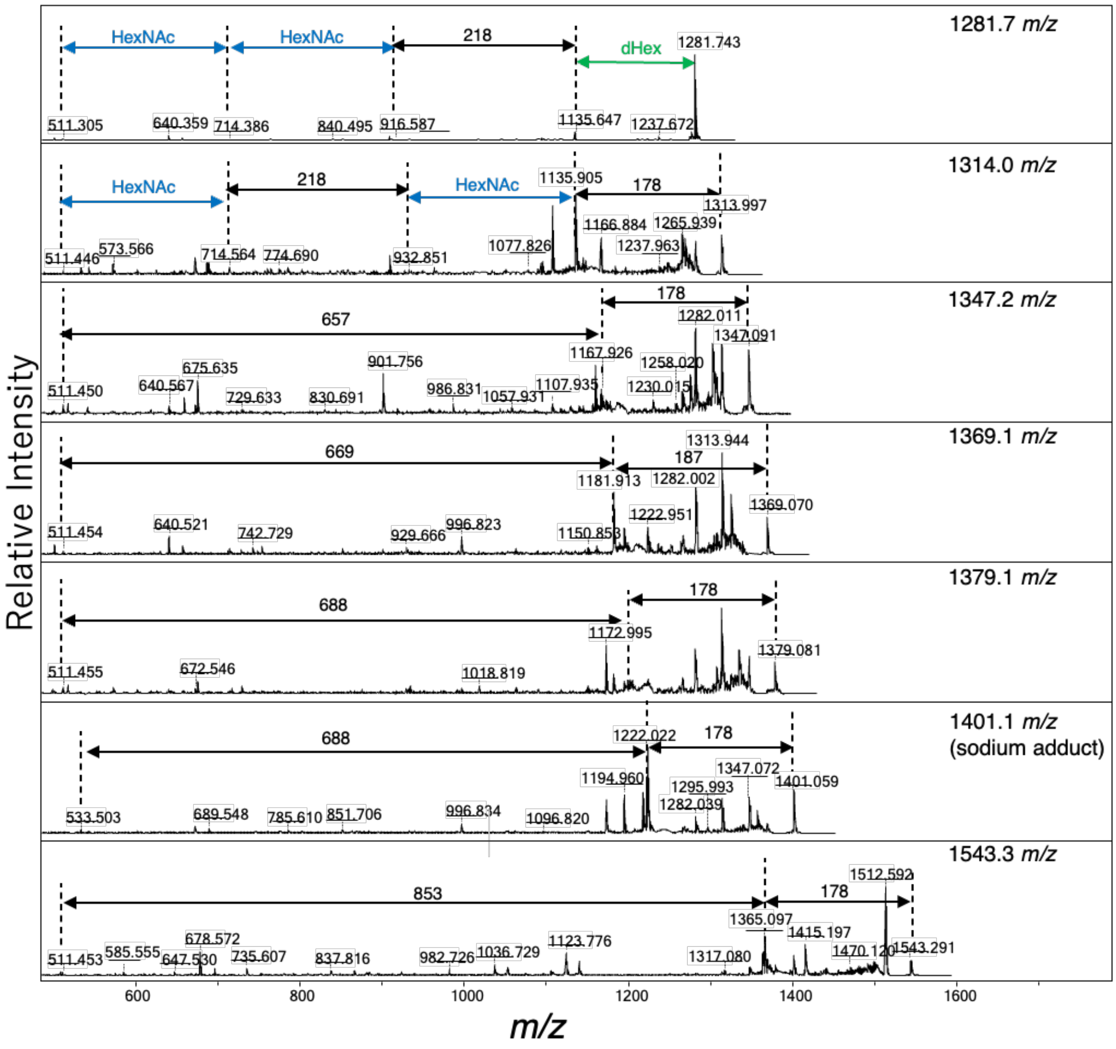

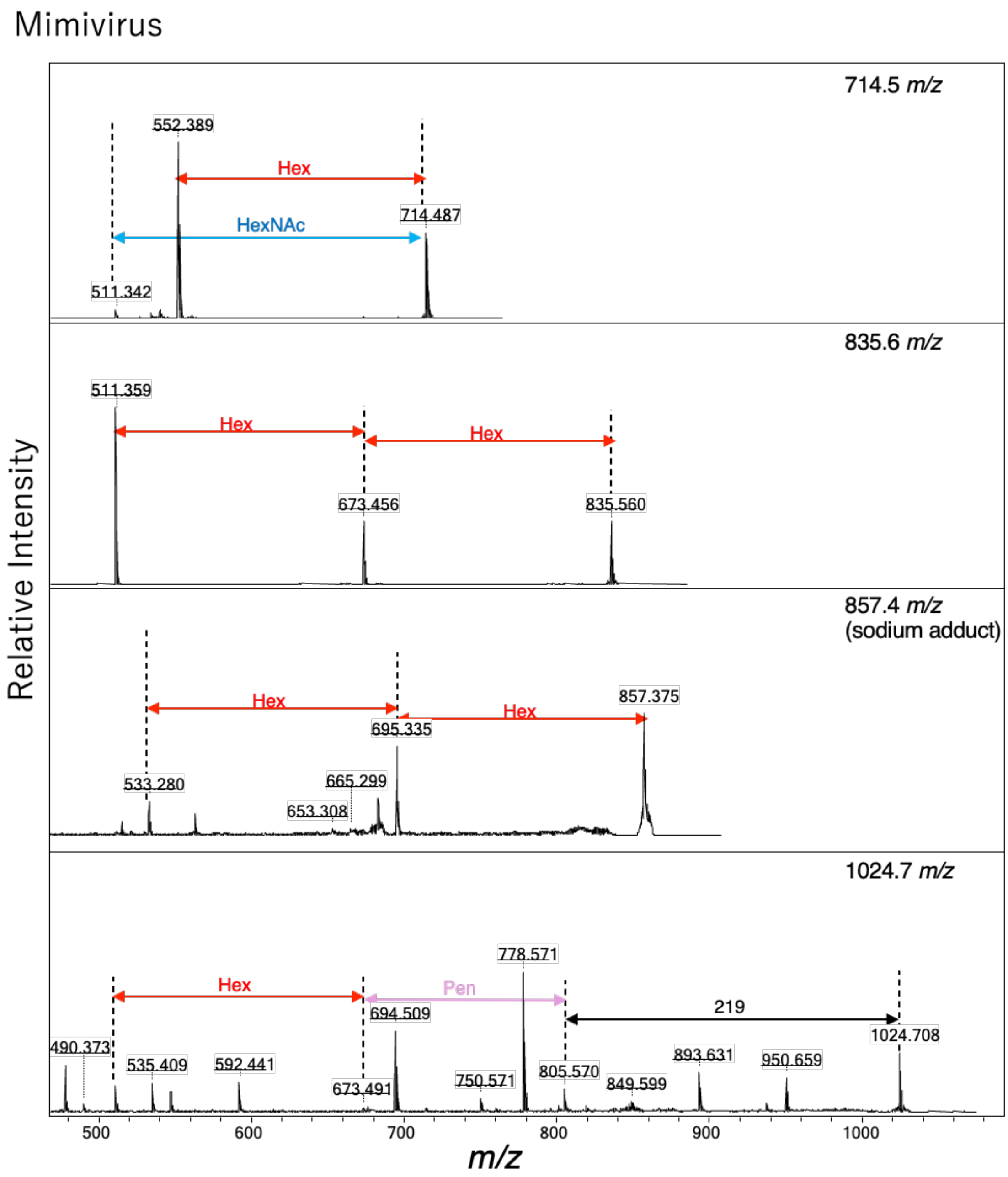

Supplementary Fig 1: MS/MS spectra of PMP-labeled *O*-glycans from host and giant viruses. Representative MS/MS spectra from *A. castellani,* tokyovirus, hokutovirus, and mimivirus are shown. Spectra were acquired in LIFT mode. Major fragment peaks are annotated with their corresponding *m/z* values. For fragment ions that could not be confidently assigned to specific monosaccharide residues, the observed mass values of the intervening peaks are indicated.

Supplementary Fig. 2:

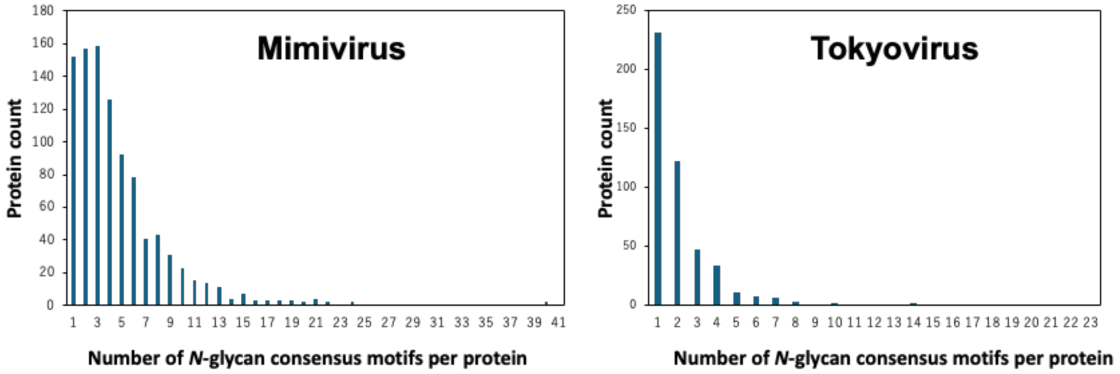

Supplementary Fig 2: Frequency of *N*-glycosylation consensus motifs per proteins in tokyovirus and mimivirus. Histograms show the distribution of consensus motifs (Asn–X–Ser/Thr, where X ≠ Pro) per protein predicted from the annotated proteomes of tokyovirus (n = 470 proteins) and mimivirus (n = 979 proteins).

Supplementary Fig. 3:

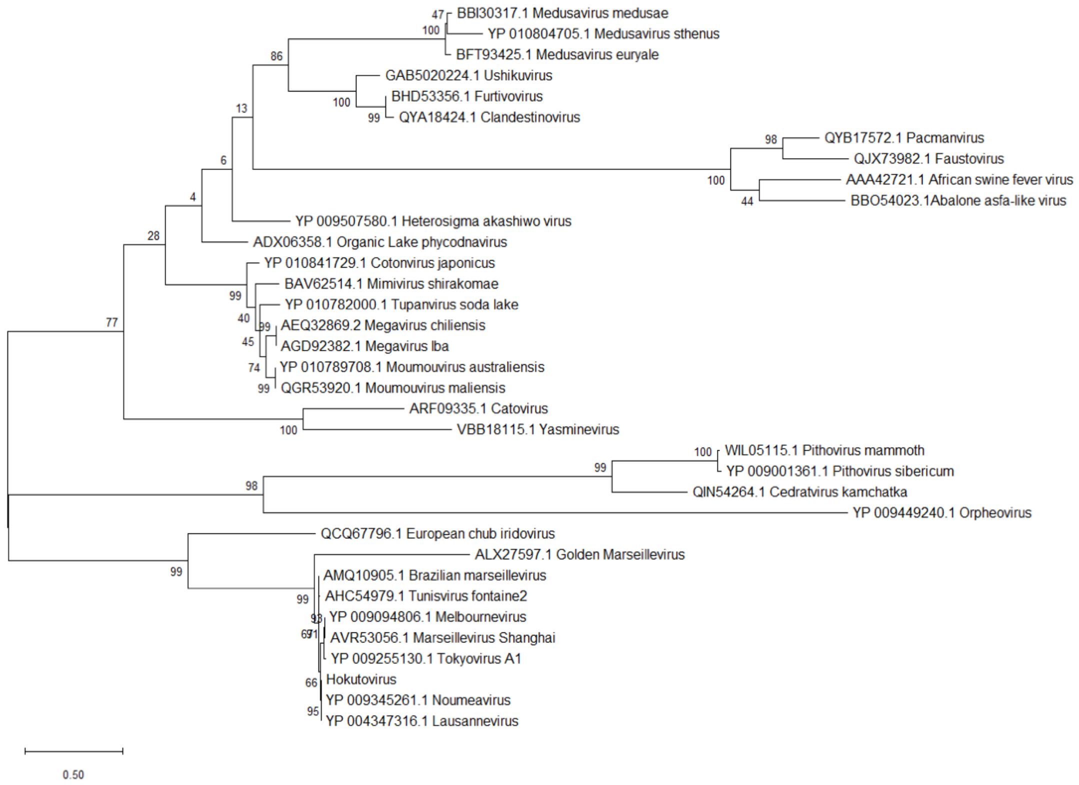

Supplementary Fig. 3: Molecular phylogenetic tree of amino acid sequences of the major capsid protein (MCP). Numbers at the branches indicate bootstrap values in 1,000 replicates. The scale bar indicates the average number of substitution per site.

Supplementary Table 1: Putative glycosyltransferase-like proteins identified in mimivirus

Gene ID	Annotation	Predicted family / domain	Putative function
**L136**	UDP-4-keto-6-deoxy-d-glucose aminotransferase	PLP-dependent aminotransferase domain	Putative amino-sugar biosynthesis (viosamine pathway)
**L137**	Glycosyltransferase family 2-like protein	GT2 family	Putative glycosyltransferase activity (GT2 type)
**L138**	Methyltransferase domain-containing protein	SAM-dependent methyltransferase	Putative sugar O-methylation
**R139**	Acetyltransferase-like protein	GNAT family	Putative N-acetylation of amino sugars
**L140**	Glycosyltransferase family 4-like protein	GT4 family	Putative glycosyltransferase activity (GT4 type)
**R141**	Glycosyltransferase family 90-like protein	GT90 family	Putative O-glycosyltransferase activity (GT90 type)
**L142**	Glycosyltransferase family 39-like protein (OCH1-like)	GT39 family	Putative α1,6-mannosyltransferase (OCH1-like activity)
**L235**	Mannosyltransferase OCH1-like protein	GT39 family	Putative α1,6-mannosyltransferase (OCH1-like activity)
**L436**	Glycosyltransferase family 2 protein	GT2 family	Putative glycosyltransferase activity (GT2 type)
**L529**	Glycosyltransferase family 4 protein	GT4 family	Putative glycosyltransferase activity (GT4 type)
**L688**	N-acetylglucosaminyltransferase-like protein	GT8 family	Putative glycosyltransferase activity (GT8 type)
**L741**	β1,3-Galactosyltransferase-like protein	GT32 family	Putative glycosyltransferase activity (GT32 type)
**L827**	Viral O-glycosylation-related glycosyltransferase	GT90 family	Putative O-glycosyltransferase activity (GT90 type)
**L1023**	Mannosyltransferase family GT17-like protein	GT17 family	Putative glycosyltransferase activity (GT17 type)
**L1115**	Fucosyltransferase-like protein	GT10 family	Putative fucosyltransferase activity (GT10 type)

Candidate genes were identified based on the mimivirus genome annotation (GenBank accession NC_014649) and domain assignments from Pfam (v35.0) and NCBI Conserved Domain Database. Glycosyltransferase-related domains were classified according to the CAZy nomenclature (GT2, GT4, GT8, GT10, GT17, GT32, GT39, and GT90).

Supplementary Table 2: Putative glycosyltransferase-like proteins identified in tokyovirus

Gene ID	Annotation	Predicted family / domain	Putative function
**TkV_167**	Mannosyltransferase OCH1-like protein 1	GT39 family	Putative α1,6-mannosyltransferase (OCH1-like activity)
**TkV_291**	Mannosyltransferase OCH1-like protein 2	GT39 family	Putative α1,6-mannosyltransferase (OCH1-like activity)
**TkV_388**	Mannosyltransferase OCH1-like protein 3	GT39 family	Putative α1,6-mannosyltransferase (OCH1-like activity)
**TkV_126**	Glycosyltransferase family 2 protein	GT2 family	Putative glycosyltransferase activity (GT2 type)
**TkV_304**	N-acetylglucosaminyltransferase-like protein	GT8 family	Putative glycosyltransferase activity (GT8 type)

Candidate genes were identified based on the functional annotation of the tokyovirus genome (GenBank accession AP017398) and domain assignments from Pfam (v35.0) and NCBI Conserved Domain Database. Glycosyltransferase-related domains were classified according to the CAZy nomenclature (GT2, GT8, and GT39).

Supplementary Table 3: Candidate *A. castellanii* genes potentially associated with unusual neutral-loss fragments (159, 178, 202, and 218 Da)

Enzyme / Function	KO ID	Role in monosaccharide biosynthesis	*A. castellanii* ORF ID
GDP-mannose 4,6-dehydratase (Gmd)	K01711	First step of perosamine biosynthesis (159 Da, 4-amino-4,6-dideoxyhexose)	ACA1_178520
PLP-dependent aminotransferase (PerA-like)	K13010	Converts 4-keto-6-deoxy-Man to 4-amino derivative	ACA1_229430
N-acetyltransferase (PerB-like)	K17939	Acetylation of amino sugar derivatives	ACA1_343210
Cytochrome P450 monooxygenases	–	Hydroxylation, generation of polyhydroxylated hexoses (178 Da candidate)	ACA1_056780, ACA1_302290, ACA1_362510
FAD-dependent oxidoreductases (GMC family)	–	Hydroxylation / redox modifications (178 Da candidate)	ACA1_112040, ACA1_244670
KdsA (3-deoxy-D-manno-oct-2-ulosonate 8-phosphate synthase)	K01627	First step in Kdo biosynthesis (218 Da candidate)	ACA1_157890
KdsC (Kdo 8-phosphate phosphatase)	K07046	Dephosphorylation in Kdo biosynthesis	ACA1_041220
KdsB (CMP-Kdo synthetase)	K00979	Activates Kdo for incorporation into glycans	ACA1_305670
